# Hypernatremia and Its Rate of Correction: The Evidence So Far

**DOI:** 10.7759/cureus.54699

**Published:** 2024-02-22

**Authors:** Sindhu C Pokhriyal, Prachi Joshi, Uma Gupta, Pulok Roy, Sunil Parkash, Kalendra Kunwar, Muthanna Mohammed Hasan Al-Ghuraibawi, Sagar Nagpal, Ruchi Yadav, Kalpana Panigrahi

**Affiliations:** 1 Internal Medicine, Interfaith Medical Center, Brooklyn, USA; 2 Public Health, Georgia Southern University, Atlanta, USA; 3 Internal Medicine, East Tennessee State University Quillen College of Medicine, Johnson City, USA; 4 Hematology and Oncology, Brookdale University Hospital Medical Center, Brooklyn, USA

**Keywords:** mortality, fluid, rate, correction, hypernatremia

## Abstract

Hypernatremia or high serum sodium levels can have many different causes, including insufficient free water intake, or excess free water losses. The management of hypernatremia focuses on resolving the underlying cause, replenishing free water deficit, and preventing further losses while closely monitoring serum sodium concentration. This systematic review was carried out using medical databases such as PubMed, PubMed Central, and Google Scholar for relevant medical literature. The identified articles were reviewed, eligibility criteria were applied, and seven research articles were identified. The effect of the rate of hypernatremia correction on both short- and long-term outcomes in volume-resuscitated patients was the focus of our search for randomized or observational studies. Based on our analysis of the clinical evidence, we concluded that the present recommendations for treating acute and chronic hypernatremia in resuscitated patients do not stem from high-quality research.

## Introduction and background

Hypernatremia at admission can be associated with a mortality as high as 70% and it accounts for as many as 1% of all the admissions from the emergency departments [1,2]. The latest recommendations on the correction of hypernatremia are largely guided by the duration of onset, i.e., acute or chronic hypernatremia. Acute hypernatremia is defined as hypernatremia that has persisted for 48 hours or less. The popular theory states that a rapid correction of sodium can induce cerebral edema and other irreversible neurological changes like osmotic demyelination syndrome that can be avoided when sodium is corrected slowly (<0.5 mmol/L/hour) [3-5]. In the current systematic review, we looked at all articles that studied the association of the rate of correction of hypernatremia with mortality and also tried to look for its association with neurological outcomes.

## Review

Methodology

The systematic review was conducted based on the Preferred Reporting Items for Systematic Reviews and Meta-Analyses (PRISMA) 2020 guidelines.

Search strategy and eligibility criteria

The present study involved a comprehensive search for relevant literature on the rate of correction of hypernatremia in hospital settings. The search was conducted using databases PubMed, PubMed Central (PMC), and Google Scholar. To ensure a focused search, exclusion criteria were applied, including consideration of only adult patients and excluding papers related to hypernatremia in head injury or pathology settings, as well as those related to COVID-19 lung pathology and hypernatremia. Papers without full-text availability and those not related to the matter of interest, i.e., the rate of correction, were also excluded. 

We researched databases like PubMed, PMC, and Google Scholar. We used various combinations of our keyword concepts, including hypernatremia, correction, rate, rapid correction, and slow correction to search all databases. In PubMed, however, along with these keywords, the following strategy was developed and used to search relevant literature in PubMed's Medical Subject Headings database (as shown in Table 1).

**Table 1:** Keywords employed in the study MeSH: Medical Subject Headings

Search strategy	Keywords
Regular keywords	Hypernatremia, correction, rate of correction, rapid correction, slow correction
MeSH keywords	("hypernatraemia"[All Fields] OR "hypernatremia"[MeSH Terms] OR "hypernatremia"[All Fields]) AND correction[All Fields] AND ("J Rehabil Assist Technol Eng"[Journal] OR "rate"[All Fields]) NOT ("animals"[MeSH Terms:noexp] OR animal[All Fields]) AND ("2014/02/05"[PDat] : "2024/02/02"[PDat])NOT ("sars-cov-2"[MeSH Terms] OR "sars-cov-2"[All Fields] OR "covid"[All Fields] OR "covid-19"[MeSH Terms] OR "covid-19"[All Fields]) AND ("2014/02/05"[PDat] : "2024/02/02"[PDat]) NOT ("infant, newborn"[MeSH Terms] OR ("infant"[All Fields] AND "newborn"[All Fields]) OR "newborn infant"[All Fields] OR "newborns"[All Fields]) NOT ("child"[MeSH Terms] OR "child"[All Fields] OR "children"[All Fields]) AND ("2014/02/05"[PDat] : "2024/02/02"[PDat])

To ensure a thorough review process, we employed three reviewers who each independently reviewed the papers selected from all the databases. The inclusion criteria for the final review were randomized controlled trials, retrospective observational studies, meta-analyses, cohort studies, narrative reviews, systematic reviews, and case series on the rate of correction of hypernatremia in hospital settings. Overall, the search and selection process was designed to provide a comprehensive and focused review of the literature on the rate of correction of hypernatremia in hospital settings.

Selection process and quality appraisal

The selection process for research articles included in our analysis was designed to ensure a focused and rigorous review of the literature on hypernatremia correction rate in acute and chronic hypernatremia patients. The selected articles were required to meet specific inclusion criteria, including the patient age range of 18-90 years, hospital settings, and no history of head injury or COVID-19 as the cause of hypernatremia. The measurement tool to assess systematic reviews was used to evaluate systematic reviews (as shown in Table 2). Overall, the selection process was carefully designed to ensure that the articles included in our analysis were relevant and provided accurate and reliable information on the hypernatremia correction rate in hospitalized patients.

**Table 2:** Newcastle Ottawa quality matrix for quality appraisal of systematic review NOS: Newcastle-Ottawa Scale

NOS items	Chauhan et al. [2]	Goshima et al. [6]	Olsen et al. [7]	Ryu et al. [8]	Feigin et al. [9]	Alshayeb et al. [10]	Bataille et al. [5]
Representativeness of the exposed cohort	0	0	1	1	1	0	0
Selection of the non-exposed cohort	0	0	1	1	1	0	0
Ascertainment of exposure	1	1	1	1	1	1	1
Demonstration that outcome of interest was not present at the start of the study	1	0	1	1	1	0	1
Comparability of cohorts on the basis of the design and analysis	1	0	1	1	2	0	1
Assessment of outcome	1	1	1	1	1	1	1
Was follow-up long enough for outcomes to occur	1	0	0	1	1	0	1
Adequacy of follow-up of cohorts	0	0	0	1	1	0	1
Total	5	2	6	8	9	2	6

Data collection

The primary findings were assessed following the extraction of the final articles for the systematic review. 

**Figure 1:**
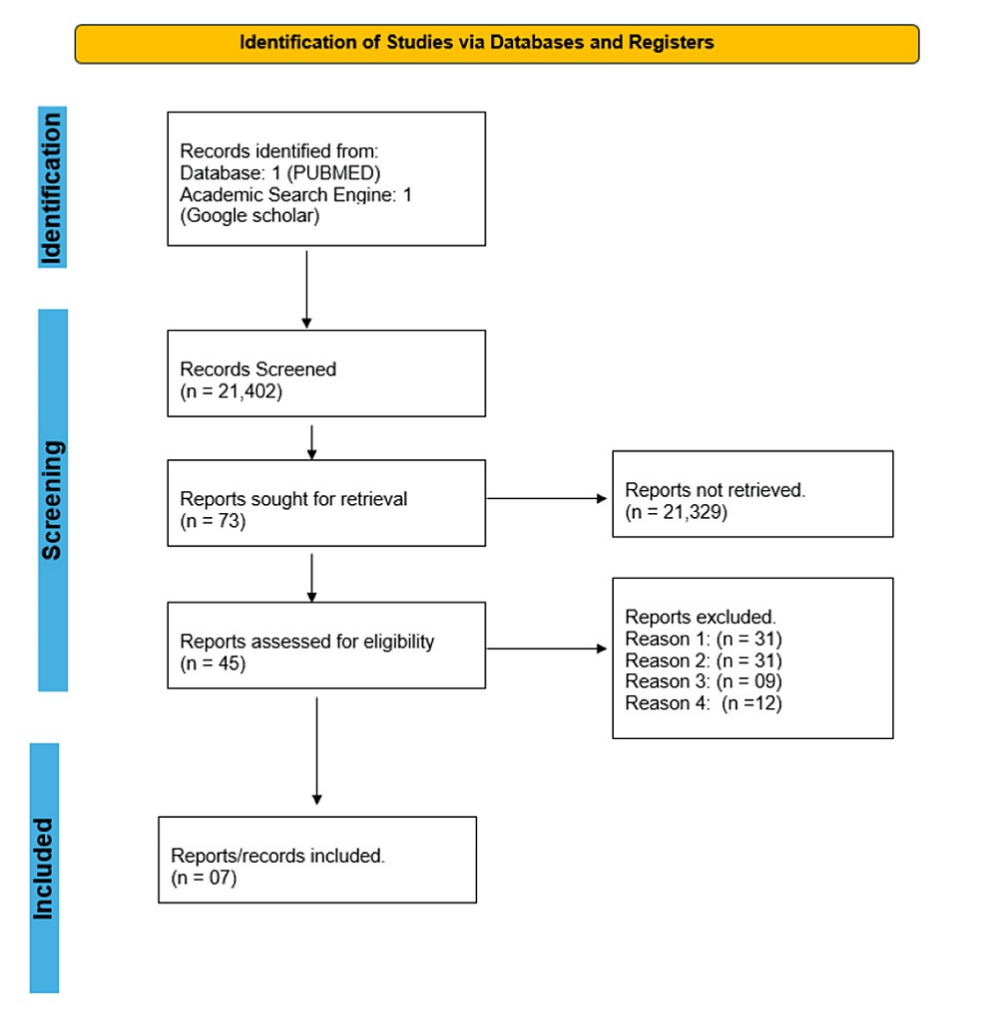
The PRISMA 2020 statement - an updated guideline for reporting systematic reviews. Keywords as given in Table 1 Reason 1: Animal studies Reason 2: Studies done on the pediatric population Reason 3: Studies on SARS-COVID-19 patients Reason 4: Studies on head injury patients

We gathered a total of 21,402 studies using all the databases. Of them, 21,329 studies were not retrieved due to irrelevance, lack of availability of full articles as well as duplication. After reviewing and screening the remaining articles based on titles, availability of full text, abstracts, and inclusion and exclusion criteria, seven published research papers were shortlisted. These shortlisted full-text papers were subjected to quality appraisal and final review; five were retrospective studies, one was an ongoing randomized controlled trial, and one was a systematic review. Figure 1 of the PRISMA flowchart shows the selection process for finalized studies.

Results

Table 3 shows the essential characteristics of the shortlisted studies.

**Table 3:** The essential characteristics of the studies included in the review

Authors/year of study	Type of study	Number of patients	Sodium levels at enrollment (mEq/L)	Rate of correction	Mortality rate	Other comments on the outcome
Chauhan et al. 2019 [2]	Retrospective observational study	327	>155	>0.5 mmol/L/hour	No difference in mortality in the rapid and slow correction group	Statistically not significant in rapid and slow correction group
Goshima et al. 2022 [6]	Retrospective observational study	18	>160	Rapid infusion of dextrose-based fluid	3 patients out of 13 had severe hypernatremia	The rapid correction group has a higher survival rate than in slow correction group
Olsen et al. 2020 [7]	Retrospective observational study	5790	Na > 145	>0.5 mmol/L/hour	Increase mortality in the rapid correction group	The rapid correction group has higher mortality which is not statistically significant
Ryu et al. SALSA II trial 2022 [8]	Prospective randomized control study	166	>155	Rapid intermittent bolus (RIB) vs slow continuous infusion (SCI)	Results awaited	Results awaited. - The limitation of the study was the complicated protocol, only electrolyte-free water was used
Feigin et al. 2023 [9]	Retrospective cohort study	4265	155	Fast correction rate vs slow correction rate	Mortality rates were significantly lower in rapidly corrected patients both at 30-day and 1-year	No evidence was identified supporting the association between rapid rate of correction and patient harm
Alshayeb et al. 2011 [10]	Retrospective	131	≥155	Effect of the rate of correction of severe hypernatremia on the mortality of hospitalized patients	30 days patient mortality rate was 37%	In patients with severe hypernatremia, the rate of correction of hypernatremia was slow, resulting in inadequate correction in most patients. Both slow rate of hypernatremia correction during the first 24 hours and do not resuscitate status were found to be significant predictors of 30-day patient mortality.
Bataille et al. 2014 [5]	Retrospective study	85	≥150	-0.24 ± 0.2 mmol/L/hour in 4.87 ± 3.28 days (from 11 hours to 14 days)	The hospital mortality rate was 24%	Not only a too-quick but also a too-slow correction rate is associated with an increased risk of death

Limitations

This study had its weakness in that only free full-version articles were reviewed. Thus, some systematic reviews might have been missed.

Discussion

Patients with hypernatremia have a mortality rate of 20% to 60%, which varies depending on comorbidities, related illnesses, and whether hypernatremia is acquired during hospitalization or present at admission. Because of this, hypernatremia is a crucial clinical entity that needs to be optimally managed to ensure favorable outcomes for patients [5,11]. The latest recommendations on the correction of hypernatremia are largely guided by the duration of onset of hypernatremia (acute vs chronic hypernatremia) and the basic protocol involves not correcting >=0.5 mmol/L/hour [1-3]. The popular theory states that a rapid correction of sodium can induce cerebral edema and other irreversible neurological changes like osmotic demyelination syndrome that can be avoided when sodium is corrected slowly [3]. However, these current recommendations on the correction of hypernatremia are mostly based on studies in children as well as in studies done on patients admitted to the intensive care units (ICU) and need to be reviewed more thoroughly [1,12].

Upon assessing the seven studies using the Newcastle-Ottawa Scale, it was determined that the studies conducted by Ryu et al. and Feigin et al. are of good quality. Chauhan et al., Olsen et al., and Bataille et al. were classified as fair quality, while Goshima et al. and Alshayeb et al. were deemed poor quality [2,6-9].

Ryu et al. stood out in analyzing the good quality studies due to their prospective study design, while Feigin et al. utilized a retrospective approach. However, Feigin et al. had a larger sample size than Ryu et al. [8,9]. Both studies addressed the theory of rapid correction of hypernatremia. Ryu et al. associated rapid correction with effective and safe outcomes, while Feigin et al. linked rapid correction to shorter hospitalization durations and significantly reduced patient mortality rates. Currently, Ryu et al. are working with two experimental groups in a prospective, multicenter, open-label, randomized controlled groups. A total of 166 individuals with severe hypernatremia have been recruited and split into two randomized groups. The first group is the rapid infusion group while the second delayed correction groups will be treated with electrolyte-free water and the major outcome measures include a rapid drop in the Na level (≥0.25 mmol/L/hour or 6 mmol/L/day) and 30-day mortality [8]. Feigin et al. in their retrospective cohort study of 4265 hypernatremia patients with a median age of 78 years observed that 343 (8.0%) had fast correction rates of hypernatremia. This cohort analysis of patients with severe hypernatremia demonstrated that prompt hypernatremia correction correlated with shorter hospitalizations and considerably lower patient mortality in the absence of neurologic sequelae [9].

Chauhan et al., Olsen et al., and Bataille et al. received fair-quality scores on the Newcastle-Ottawa Scale [2,5,7]. Chauhan et al. conducted a retrospective study with critically ill ICU patients, which may not represent the overall population. After excluding individuals without follow-up serum sodium values above their peak level, 449 patients were included in the main study. They further divided the patients into two groups; of which 122 had severe hypernatremia at admission, and 327 had hospital-acquired hypernatremia. The 30-day survival was analyzed using Kaplan-Meier curves for the fast and slow correction rates. Analysis was done on all patients to determine the incidence of in-hospital mortality by fast versus slow correction rates using various cut-offs (>8, >10, and >12 mmol/L in 24 hours). Additionally, 30-day survival estimates were essentially identical across the board for all patients, with some quick correction rate groups showing a trend toward lower mortality that did not statistically significantly differ. Furthermore, according to subgroup analyses, patients who had acute hypernatremia in a group characterized by a 48-hour interval had a greater mortality proportion than patients who had hospital-acquired hypernatremia (47% versus 32%; p=0.01). In any duration of hypernatremia development, there was no discernible difference in the mortality proportion between the groups receiving faster and slower correction [2]. The study's findings aligned with those of Feigin's high-quality study on the Newcastle-Ottawa Scale [2,9]. The study did not find evidence to support the claim that rapid correction of hypernatremia is associated with a higher risk of mortality or other conditions, such as seizures, altered consciousness, and cerebral edema, in critically ill patients with either admission or hospital-acquired hypernatremia [2,9].

Olsen et al. conducted a retrospective study with a representative sample size. The study observed two cohorts and utilized the ICU patient database to record observations. However, the study did not mention follow-up records or the frequency of follow-ups. The study's results contradicted Feigin's high-quality studies, concluding that ICU-acquired hypernatremia is linked to increase in-hospital mortality and suggesting that rapid sodium correction rates may potentially harm ICU patients. Olsen et al. in their study sought to investigate the optimal hypernatremia correction rate amongst two cohorts of ICU patients namely the Medical Information Mart for Intensive Care III and the electronic ICU patients. Each cohort had 2172 and 5790 patients, respectively, who developed hypernatremia during their ICU stay, respectively. In both cohorts, a rapid sodium correction rate (>0.5 mmol/L/hour) was linked to higher in-hospital mortality (odds ratios, 1.08; 95% CI, 1.03-1.13 for Medical Information Mart for Intensive Care III and 1.10; 95% CI, 1.06-1.13) but the results were statistically significant only in the electronic ICU group. Thus, they concluded that rapid sodium correction rates may be harmful. Using existing literature-based thresholds, the patients' sodium correction rates were divided into three groups: slow (<0.25 mmol/L/hour), moderate (0.25-0.50 mmol/L/hour), and fast (>0.50 mmol/L/hour) correction of serum sodium. To describe the impact of peak sodium, hypernatremic duration, and hypernatremic load on in-hospital mortality, they employed univariable logistic regression. The Wilcoxon signed-rank test was used to perform subgroup analyses for the hypernatremic groups and sodium correction rates. P values are only shown following Bonferroni correction. R 4.0.0 was used to analyze the data (R Core Team, Vienna, Austria). This study possibly underlines the importance of conducting bigger randomized controlled trials to investigate the efficacy of various correction rates in individuals with hypernatremia [7].

Bataille et al. scored the highest among the fair-quality studies. It can be considered a study of intermediate quality. This retrospective study was conducted on all patients admitted to the Center of Marseille, France ED from 2010 to 2011. The study's robust and representative sample size provides an advantage. The study claims that slow correction speed is associated with an increased risk of death regardless of initial natremia, supporting the claims of high-quality studies and contradicting the findings of Olsen et al. Bataille et al. reviewed 85 cases of severe hypernatremia ≥150 mmol/L who had been admitted to ED. Among the 85 patients, 78 (92%) required hospitalization, 5 went back to their prior facility, 1 passed away in the ED, and 1 patient's prognosis was unknown. They were unable to examine the relationship between natremia correction speed and perfusion speed since 74% of the sample did not know what their perfusion speed was. After accounting for this limitation, a multivariate analysis revealed that a slow mean correction speed to the last known natremia was linked with higher in-hospital mortality (p < 10-3; HR = 10.29 95% CI (3.12-33.96)) [5].

Goshima et al. and Alshayeb et al. scored poorly on the Newcastle-Ottawa Scale. Goshima et al. employed a case series design, which is not considered the strongest form of evidence. Case series studies often lack quality due to the absence of a control group and the potential for bias. Although the results of this study align with those of high-quality studies and conclude that rapid correction of hypernatremia leads to higher survival rates, the conclusion is based on only 18 case reports. Goshima et al. in their retrospective study included 18 patients with a median sodium level of 181.5. They conducted an estimation of the correction rates for all paired sodium levels that were recorded at two consecutive periods at least 30 minutes apart. They observed that the correction rates were more rapid in 13 successfully treated patients than in 5 fatal patients. The successfully treated patients typically achieved (Na) ≤160 within 8 hours, (Na) ≤150 within 24 hours, and (Na) ≤145 within 48 hours. However, the small sample size impacts the study's quality and weakens the evidence supporting the conclusion [6].

Alshayeb et al.'s study is of poor quality due to limited accessibility to the article. It is a retrospective study with a sample size of 131 patients. They found that in patients with severe hypernatremia of >159meQ/L, slow correction of hypernatremia in the first 24 hours of <0.25mmol/L was an independent risk factor for increased 30-day patient mortality rate. They also found that patients whose hypernatremia was corrected within 72 hours had significantly lower mortality rates when compared to their other counterparts (10% versus 25%). However, the study's results claim that slow correction of hypernatremia is a predictor of 30-day patient mortality. Although the study aligns with high-quality studies, the fact that the results are derived from chart reviews weakens the study design and diminishes its support for the concluding claims. Also, another major limitation was that they deemed sodium correction > 0.134mmol/L/hour as a rapid correction rate [10].

Hyponatremia and its rate of correction might be difficult to address in large-scale prospective studies on patients presenting to the hospital with hypernatremia. So, although case reports and series, might not use the best clinical research methodology, they are an effective way to supplement existing evidence with facts. The creation and study of a prospective, international consortium registry of this patient group could be another realistic way to find out more about prognostic factors that predict which patients won't respond well to certain regimens, including the recommended treatments, and help develop risk-adapted sodium correction strategies. We believe that while we eagerly await the results of the SALSA II trial, the above strategy too might help us develop risk-adapted sodium correction strategies. Finally, there is a need for studies that focus more on neurological outcomes along with mortality, as neurological sequelae are said to represent the direct outcome of rapid correction of hypernatremia especially when it is chronic.

## Conclusions

The results of our study suggest that there is no strong scientific evidence at present to strongly substantiate the current practices and recommendations in the rate of correction of hypernatremia. In different clinical contexts, rapid correction of hypernatremia may not be as harmful as it is deemed to be. Finally, more good quality studies are needed to study the effect of the rate of correction of hypernatremia on mortality rates as well as neurological events in patients admitted with both acute as well as chronic hypernatremia.
